# Evaluation of Candidate Reference Genes for Normalization of Quantitative RT-PCR in Soybean Tissues under Various Abiotic Stress Conditions

**DOI:** 10.1371/journal.pone.0046487

**Published:** 2012-09-28

**Authors:** Dung Tien Le, Donavan L. Aldrich, Babu Valliyodan, Yasuko Watanabe, Chien Van Ha, Rie Nishiyama, Satish K. Guttikonda, Truyen N. Quach, Juan J. Gutierrez-Gonzalez, Lam-Son Phan Tran, Henry T. Nguyen

**Affiliations:** 1 Signaling Pathway Research Unit, RIKEN Plant Science Center, Yokohama, Kanagawa, Japan; 2 National Center for Soybean Biotechnology and Division of Plant Sciences, University of Missouri, Columbia, Missouri, United States of America; Kyushu Institute of Technology, Japan

## Abstract

Quantitative RT-PCR can be a very sensitive and powerful technique for measuring differential gene expression. Changes in gene expression induced by abiotic stresses are complex and multifaceted, which make determining stably expressed genes for data normalization difficult. To identify the most suitable reference genes for abiotic stress studies in soybean, 13 candidate genes collected from literature were evaluated for stability of expression under dehydration, high salinity, cold and ABA (abscisic acid) treatments using delta CT and geNorm approaches. Validation of reference genes indicated that the best reference genes are tissue- and stress-dependent. With respect to dehydration treatment, the *Fbox*/*ABC*, *Fbox*/*60s* gene pairs were found to have the highest expression stability in the root and shoot tissues of soybean seedlings, respectively. *Fbox* and *60s* genes are the most suitable reference genes across dehydrated root and shoot tissues. Under salt stress the *ELF1b*/*IDE* and *Fbox*/*ELF1b* are the most stably expressed gene pairs in roots and shoots, respectively, while *60s*/*Fbox* is the best gene pair in both tissues. For studying cold stress in roots or shoots, *IDE*/*60s* and *Fbox*/*Act27* are good reference gene pairs, respectively. With regard to gene expression analysis under ABA treatment in either roots, shoots or across these tissues, *60s*/*ELF1b*, *ELF1b*/*Fbox* and *60s*/*ELF1b* are the most suitable reference genes, respectively. The expression of *ELF1b*/*60s*, *60s*/*Fbox* and *60s*/*Fbox* genes was most stable in roots, shoots and both tissues, respectively, under various stresses studied. Among the genes tested, *60s* was found to be the best reference gene in different tissues and under various stress conditions. The highly ranked reference genes identified from this study were proved to be capable of detecting subtle differences in expression rates that otherwise would be missed if a less stable reference gene was used.

## Introduction

The study of plant adaptive responses to abiotic stresses, such as drought, high salinity and cold stress, is a rapidly growing field of research due largely to its immense impact on global food supply. Abiotic stressors are the adverse environmental conditions which are unfavorable to plant growth and include such circumstances as flooding, extreme temperatures, high soil salinity, and drought. These stressors can have detrimental effects on plants which generally result in major yield losses for the economically important crops, including soybean [1]–[5]. Understanding the mechanisms plants use to cope with such stresses is crucial for areas of research aimed at the engineering of soybean cultivars with increased stress tolerance [6]–[9]. Of the various environmental stresses, drought is one which typically receives much attention due to its pervasiveness as well as the significance of its impact to soybean yields worldwide [3], [10].

Plants have always evolved under the highly selective pressure of these stresses and have thus established exceedingly complex and broad-stroked genetic and molecular mechanisms to survive the adverse conditions imposed by them. It has been demonstrated in many instances that many of the primary mechanisms plants use to cope with abiotic stress are not constitutively active throughout their lifetime, but are induced at a transcriptional level when these stresses are present [6], [9], [11]. Therefore, much of the research related to abiotic stress is focused on determining the key factors involved in such responses, and elucidating the network of genes induced and/or regulated by these factors is one of the most powerful approaches researchers can employ to study stress tolerance [9], [12]–[18]. Consequently, the ability to perform high-throughput profiling sensitive enough to detect subtle changes in the expression levels of a magnitude of target genes is a valuable tool for research focused on abiotic stress-induced gene expression [1], [10], [19].

Three profiling techniques are currently available to study the changes in gene expression induced by abiotic stress factors. Northern blot is the first technique to have emerged which offered the capability to measure differential gene expression. This technique typically involves using electrophoretically separated RNA which is then transferred to a membrane and hybridized with a detectable complementary probe [20]. But northern blots are both time consuming and labor intensive. And although the sensitivity threshold is quite high, the qualitative nature of the technique limits the ability to accurately quantify expression levels. This, in conjunction with the low throughput aspect of northern blotting, restricts its effectiveness for expression profiling the magnitude of target genes often required for abiotic stress studies. The emergence of DNA microarray hybridization, using either cDNA microarrays or oligonucleotide microarrays [21]–[23], has enabled the ability to effectively screen a large number of target genes under various conditions, including environmental stimuli [24]–[29]. This technology uses an arrayed series of microscopic spots of DNA sequences representing individual target genes [30]. For expression profiling studies using two-color microarray, template cDNAs from control and treated samples labeled with different fluorophores are hybridized to the array, and the fluorescent signals from both fluorophores are determined for each probe spot. The differences between signal intensities from each fluorophore are used to determine the changes in gene expression between the control and treatment samples [31]. On the other hand, one-color microarrays provide intensity data for each probe which together indicate a relative level of hybridization with the labeled target [32]. The newly emerging whole-genome tiling arrays covering whole genome sequence of both strands with oligo probes have allowed us to identify many stress-inducible genes and transcripts including non-protein-coding RNAs, which were not able to be unidentified by the use of cDNA microarrays and oligonucleotide microarrays [33], [34]. Although all of these microarray platforms enable us to screen thousands of gene probes simultaneously, microarray technology is not sensitive enough for detecting modest changes in gene expression [35]. Additionally, the specificities of the probe oligonucliotides in these arrays are not always high enough to ensure that individual genes are being hybridized.

Quantitative real-time polymerase chain reaction (qRT-PCR) is potentially the most sensitive method developed for detecting the changes in gene expression [36]. This process uses fluorescent dyes or probes to detect the amount of double-stranded DNA amplified from template DNA during a series of sequential PCR reactions using forward and reverse primer sequences designed to amplify a segment of a particular target gene [37], [38]. The fractional PCR cycle number at which this fluorescent signal achieves a defined threshold is referred to as the Ct value and is proportional to the abundance of that target gene in the template DNA. Like DNA microarray hybridization, qRT-PCR has the ability to perform screening of a multitude of gene targets. However, unlike DNA microarrays, the nature of the PCR reaction intrinsic to this method allows for both a high sensitivity of detection as well as the use of high specificity primers to ensure that the expression levels of individual genes are being measured. These aspects of qRT-PCR make it an attractive tool for use in research related to studying the changes in gene expressions induced by abiotic stresses in plants [23], [39]–[42].

However, as powerful and sensitive as qRT-PCR has the potential to be, it is not without its pitfalls. A fundamental constraint on the accurate interpretation of qRT-PCR data is the necessity of a stable, constitutively-expressed reference gene which can be used as a standard measure for comparing one sample to another [43]. This normalization is typically performed in order to compensate for the variability between samples contributed by the multitude of factors which can influence the cycle threshold (CT) value obtained from a qRT-PCR reaction. Ideal reference genes for the normalization of qRT-PCR data are genes which demonstrate a consistent level of expression among control and treated samples, and in the context of studies under stresses, the expression levels of these reference genes would be unaffected by the stress treatments applied and would allow for the accurate normalization of target gene expression levels between samples. The changes of gene expression levels induced by abiotic stresses like drought, high salinity and cold can be complex and multifaceted, often affecting the expression levels of genes which would otherwise be stable and suitable as reference genes in other experimental circumstances. Consequently, it is crucial that the expression stability of potential reference gene be confirmed under abiotic stress conditions before they are utilized for the normalization of qRT-PCR data generated in these studies. For soybean, several reference genes have been available; however, these genes were validated only under normal or biotic stress conditions [44]–[46]. An extended search in literature identified an article on the journal of *Pesquisa Agropecuária Brasileira* by Stolf-Moreira and coworkers which described the search for reference genes for use in qRT-PCR analysis of soybean under drought condition only [47]. Therefore, the primary objective of this study was to determine in systematic manner reference genes which demonstrate a high degree of expression stability under various abiotic stress conditions in soybean, to facilitate a more accurate normalization of qRT-PCR assays.

## Materials and Methods

### Plant materials, growth conditions and treatments

Soybean cv. Williams 82 seeds were germinated in 6-litre pots containing vermiculite soil and grown under greenhouse conditions (continuous 30°C temperature, photoperiod of 12 h/12 h, 80 µmol m−2 s−1 photon flux density and 40–60% relative humidity). For non-stress treatment all the plants were watered at regular intervals. For stress treatment, 12-d-old plants were carefully removed from soil, and roots were gently washed to remove soil. Dehydration treatment was performed as described in [40]. For NaCl and ABA (abscisic acid) treatments, 12-d-old seedlings were immersed in a solution containing either 200 mm NaCl and 100 µm ABA, respectively, for 0, 2, and 10 h. Cold treatment was performed by transferring 12-d-old plants to a container maintained at 4°C for 0, 2 and 10 h. A set of plant samples was also maintained in water at room temperature for the same durations as control. After the treatments, root and shoot samples were separately collected in three biological repeats for expression analyses.

### RNA isolation, DNAse treatment, and cDNA synthesis

Total RNA was isolated as described by using TRIZOL reagent (Invitrogen) according to the protocol provided by the manufacturer. RNA concentration and integrity were measured prior to DNase digestion with the NanoDrop UV-Vis spectrophotometer (NanoDrop Technologies). For each sample, 4 µg of total RNA was digested in a volume of 25 µl with Turbo DNA-free DNase I (Ambion). First-strand cDNA synthesis was performed using 1 µg of DNase I-treated RNA with the ReverTra Ace® qPCR RT Kit (Toyobo, Japan) in a 20-µl reaction volume according to the manufacturer's supplied protocol. All procedures were performed essentially as previously reported [40].

### qRT-PCR and data analyses

qRT-PCRs were performed in 96-well plates on a Stratagene MX3000P system (Agilent Technologies, Santa Clara, CA, USA) using Thunderbird™ SYBR® qPCR Mix (Toyobo, Japan). Primer sets (0.4 µM final concentrations for each primer) were used in a final volume of 10 µl per well. The thermal profile of the qRT-PCRs was at 95°C for 1 min, 40 cycles at 95°C for 15 s and at 60°C for 1 min. Dissociation curves (Figure S1) were obtained using a thermal melting profile performed after the last PCR cycle: 95°C for 15 s followed by a constant increase in the temperature between 60°C and 95°C. Background-corrected raw fluorescence data were exported from the MX3000P system and analyzed in LinRegPCR software with a built-in baseline correction and amplification efficiency calculation [48], [49]. Amplicon-based fluorescence thresholds were used to obtain the CT values. For confirmation of primer specificity, amplicon length was verified by electrophoresis of products through a 2% agarose gel (data not shown). Delta CT analyses were performed essentially as described by Silver et al. [50]. The mean of standard deviations of delta CTs was used to rank the performance of each candidate reference gene. The lower the values are, the more stable the expression of the candidate genes is (Table S1).

## Results and Discussion

### Screening of universal candidate reference genes for dehydration, high-salinity, cold and ABA treatments

The strategy conceived for determining effective reference genes in soybean began with a screening of the candidate genes whose expression were commonly stable across the major stressors, including dehydration, salt and cold stresses, which are often encountered by soybean plants [1], [3]. Gene expression in response to abiotic stress has been known to be regulated in ABA-dependent and/or ABA-independent manner [39], [51]–[53]; thus, we also included ABA treatment into our study. A qRT-PCR assay was designed to measure the expression stability between control and stress- or ABA-treated samples of thirteen candidate reference genes obtained from published literature related to qRT-PCR expression profiling in soybean (Table 1, Dataset S1, Figure S1). These candidate genes were chosen based on their functional homology to genes that appear to demonstrate high expression stability in other plant systems [44], [45], [54]. Additionally, candidate genes were chosen which have been previously shown to demonstrate high stability in soybean under biotic stress conditions [46]. The *18S* ribosomal RNA gene was initially included among candidate reference genes for testing but was later excluded due to its extremely high abundance. Template cDNA for the samples analyzed with *18S* primers had to be diluted at least 1000-fold relative to all other candidate genes to avoid the signal saturation obtained at low CT values. This dilution introduces a random element of variability which can potentially alter the apparent expression levels of target genes normalized with *18S*.

**Table 1:** List of primer sequence and related information for each candidate reference gene.

Genes	Functions	Glyma ID	Forward (5′→3′)^a^	Reverse (5′→3′)^a^	Amplicon length (nt)	Amplification efficiencies^b^	References
*60s*	60s Ribosomal protein L30	Glyma17g05270	AAAGTGGACCAAGGCATATCGTCG	TCAGGACATTCTCCGCAAGATTCC	125	1.910	[54]
*ABC*	ATP-binding cassette transporter	Glyma12g02310	GATCAGCAATTATGCACAACG	CCGCCACCATTCAGATTATGT	106	1.866	[44]
*Act11*	Actin	Glyma18g52780	CGGTGGTTCTATCTTGGCATC	GTCTTTCGCTTCAATAACCCTA	142	1.873	[46]
*Act27*	Actin	Glyma19g32990	CTTCCCTCAGCACCTTCCAA	GGTCCAGCTTTCACACTCCAT	119	1.858	[46]
*CDPK*	CDPK-related protein kinase	Glyma10g38460	TAAAGAGCACCATGCCTATCC	TGGTTATGTGAGCAGATGCAA	97	1.885	[44]
*CYP2*	Cyclophilin	Glyma12g02790	CGGGACCAGTGTGCTTCTTCA	CCCCTCCACTACAAAGGCTCG	154	1.855	[46]
*ELF1a*	Eukaryotic elongation factor 1 alpha	Glyma19g07240	GACCTTCTTCGTTTCTCGCA	CGAACCTCTCAATCACACGC	195	1.824	[46]
*ELF1b*	Eukaryotic elongation factor 1 beta	Glyma02g44460	GTTGAAAAGCCAGGGGACA	TCTTACCCCTTGAGCGTGG	118	1.870	[46]
*Fbox*	F-box protein family	Glyma12g05510	AGATAGGGAAATTGTGCAGGT	CTAATGGCAATTGCAGCTCTC	93	1.883	[44]
*IDE*	Insulin-degrading enzyme	AW310136	ATGAATGACGGTTCCCATGTA	GGCATTAAGGCAGCTCACTCT	131	1.884	[44]
*SUBI2*	Ubiquitin	Glyma13g17830	AGCTATTCGCAGTTCCCAAAT	CAGAGACGAACCTTGAGGAGA	84	1.862	[44]
*TUBa*	Tubulin	Glyma05g29000	AGGTCGGAAACTCCTGCTGG	AAGGTGTTGAAGGCGTCGTG	159	1.861	[46]
*TUBb*	Tubulin	Glyma20g27280	CCTCGTTCGAATTCGCTTTTTG	CAACTGTCTTGTCACTTGGCAT	161	1.844	[46]

a The exact locations of primers on the transcripts are shown in the data set S1.

b As calculated by LinRegPCR software.

**Figure 1:**
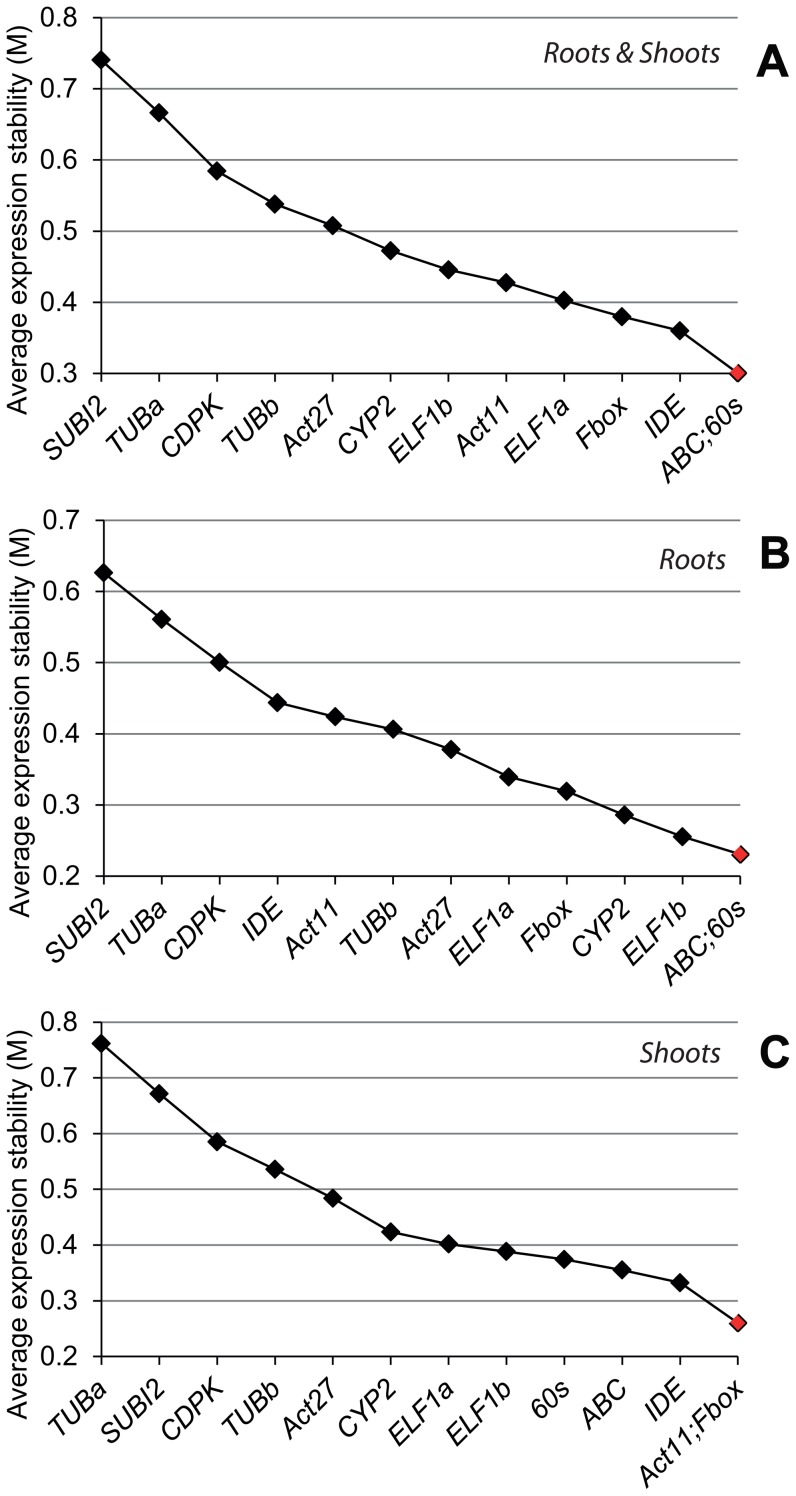
Expression stability of the candidate reference genes in root and shoot tissues of soybean seedlings under various abiotic stress and hormonal treatments. Soybean seedlings were subjected to dehydration, cold stress, salt stress and ABA treatments, and geNorm was used to assess expression stability in (A) treated roots and shoots; (B) treated roots; (C) treated shoots.

To identify reference genes that can be widely used in gene expression analyses under various stresses, the data for all stress treatments were analyzed together for root and shoot tissues of young soybean seedlings by two methods: the delta CT and geNorm. These two types of tissues are preferably used for high-throughput gene expression profiling by qRT-PCR under abiotic stresses [39]–[41], [55]–[58]. As shown in Table 2, data analysis using the delta CT method suggested that the five most stable genes in both root and shoot tissues under normal and stress conditions were *60s*>*Fbox*>*ELF1b*>*ABC*>*IDE*. When the same datasets were analyzed using geNorm, a method used to determine gene expression stability (M) [59], four of the above five genes were also among the top five most stably expressed genes although the exact order was different (*60s*/*ABC*>*IDE*>*Fbox*>*ELF1a*) (Figure 1A).

**Table 2:** Cycle thresholds and average of standard deviations (STDEVP) of delta CT obtained from tissues under all stress combinations.

	Mean CT		STDEVP of CT		Average of STDEVP of ΔCT		
	Roots	Shoots	Roots	Shoots	Roots	Shoots	Roots & Shoots
*60s*	21.31	21.97	0.5507	0.8184	***0.4274***	***0.5112***	***0.4492***
*ABC*	23.64	24.38	0.5522	0.6079	**0.4580**	0.5851	**0.5238**
*Act27*	21.64	22.03	0.5793	1.0963	0.5325	0.6736	0.5837
*Act11*	19.11	19.70	0.7787	0.7892	0.5772	**0.5761**	0.5682
*CDPK*	25.79	26.35	0.8712	0.8752	0.7810	0.8356	0.8015
*CYP2*	17.63	17.68	0.5748	0.5359	**0.5148**	0.5897	0.5987
*ELF1a*	18.41	18.87	0.5853	0.7598	0.5210	0.5905	0.5541
*ELF1b*	20.67	21.86	0.5545	0.7702	***0.4204***	**0.5462**	**0.5233**
*Fbox*	21.09	21.55	0.5217	0.6606	**0.4908**	***0.5115***	***0.4843***
*IDE*	21.66	22.41	0.7632	0.6132	0.5284	**0.5518**	**0.5333**
*SUBI2*	25.30	26.65	1.1050	0.8995	0.9455	1.1124	1.0802
*TUBa*	20.12	20.34	0.6796	1.0552	0.8019	1.1125	0.9594
*TUBb*	20.60	21.16	0.6805	1.0289	0.5258	0.7495	0.6293

Data obtained for the top five genes are shown in bold letters, while those for the top two genes are in italic and bold letters.

In real situation, we often study the differential expression of genes in a specific tissue rather than among various tissues. Thus, we subsequently looked for reference genes that performed well in individual tissues (roots or shoots) under various stresses. Analysis of the data using the delta CT method indicated that the most stably expressed genes in the root and shoots tissues are not the same, although they are overlapping (Table 2). For example, we found that *ELF1b*>*60s*>*ABC*>*Fbox*>*CYP2* and *60s*>*Fbox*>*ELF1b*>*IDE*>*Act11* constituted the top five most stable genes in the roots and shoots, respectively. By geNorm method, the top five most stable genes for roots and shoots are *ABC*/*60s*>*ELF1b*>*CYP2*>*Fbox* and *Act11*/*Fbox*>*IDE*>*ABC*>*60s*, respectively (Figures 1B and 1C). The gene expression stability (M) of the reference genes determined by geNorm was developed by Vandesompele et al. [59]. This method is based on the assumption that the expression ratio of two ideal reference genes remains constant in all samples and is unaffected by treatment conditions. Genes having the lowest M value are considered to be the most stable, while those with higher M value indicate less expression stability. The two methods of reference gene analysis have provided similar conclusions, thereby strengthening the legitimacy of the results obtained.

### Evaluation of reference gene stability in soybean root and shoot tissues under dehydration stress

**Figure 2:**
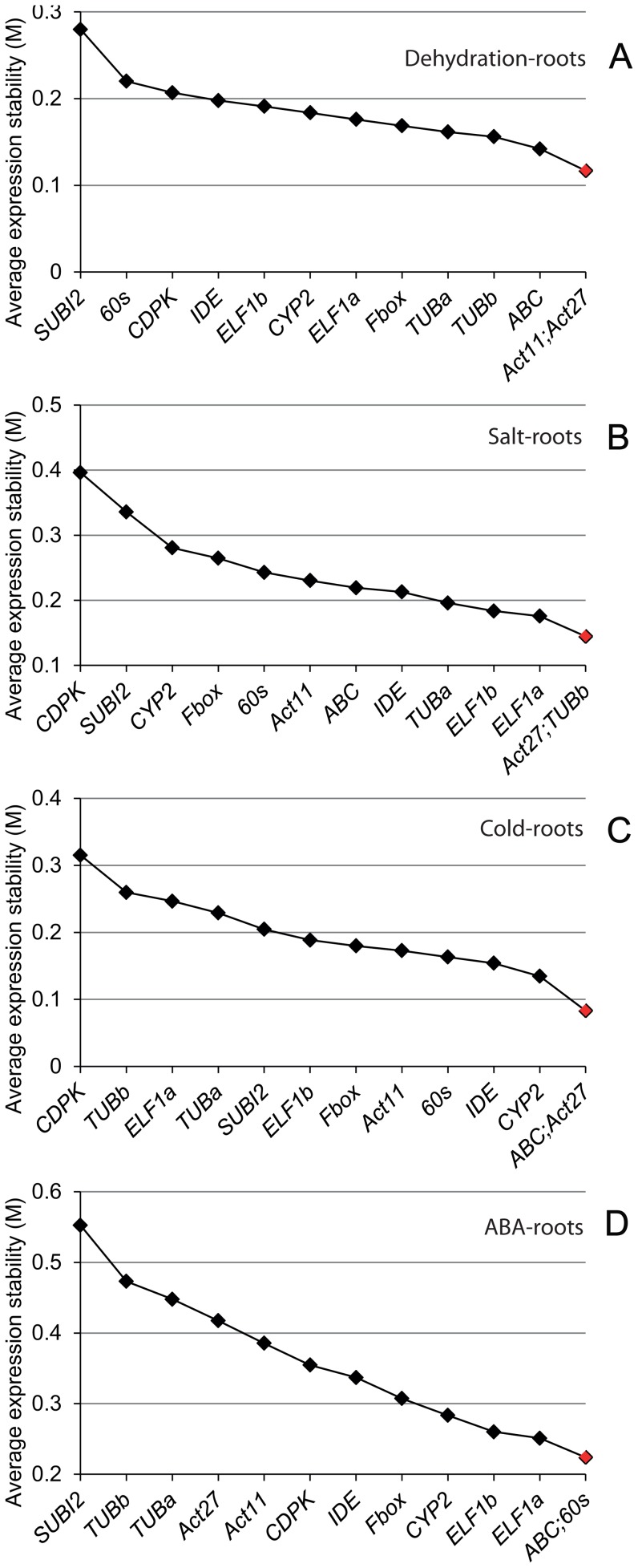
Expression stability and ranking for the candidate reference genes as determined by geNorm in the root tissues under individual stress or hormonal treatment. (A) Dehydration treatment. (B) Salt stress treatment. (C) Cold stress treatment. (D) ABA treatment.

**Figure 3:**
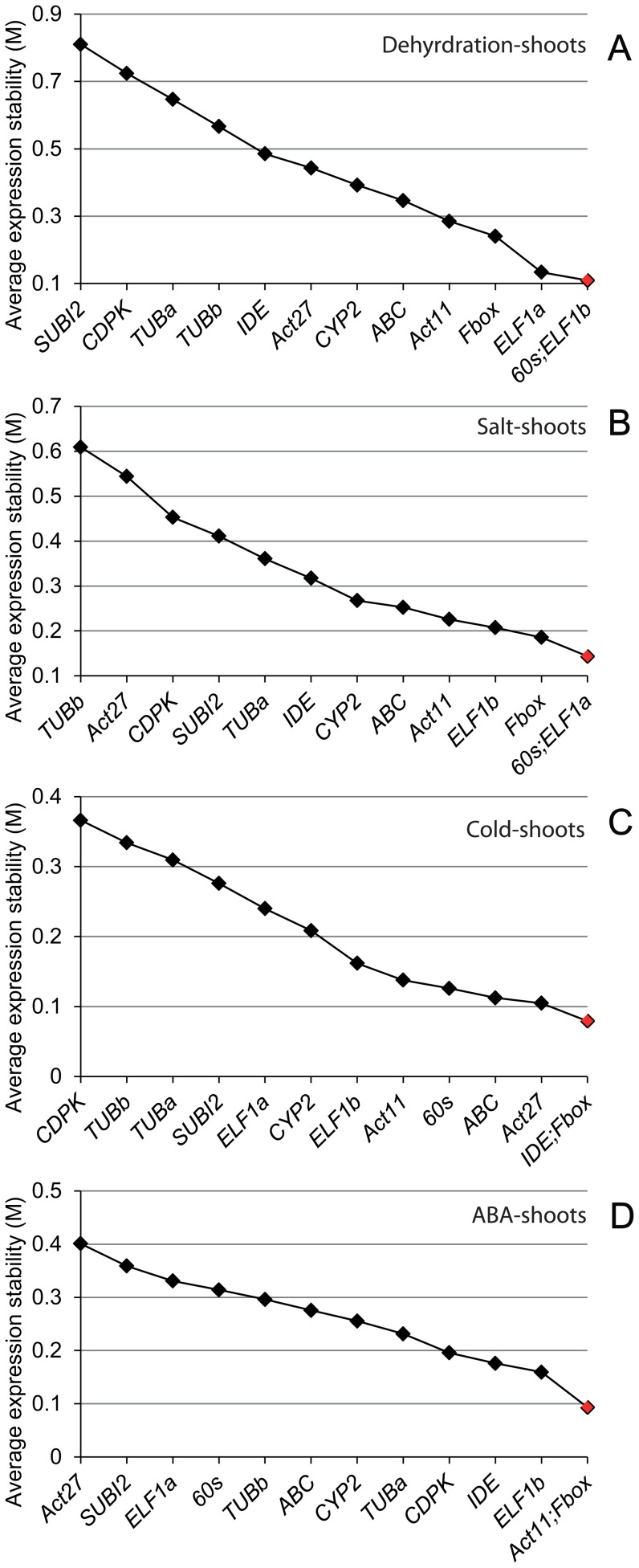
Expression stability and ranking for the candidate reference genes as determined by geNorm in the shoot tissues under individual stress or hormonal treatment. (A) Dehydration treatment. (B) Salt stress treatment. (C) Cold stress treatment. (D) ABA treatment.

**Figure 4:**
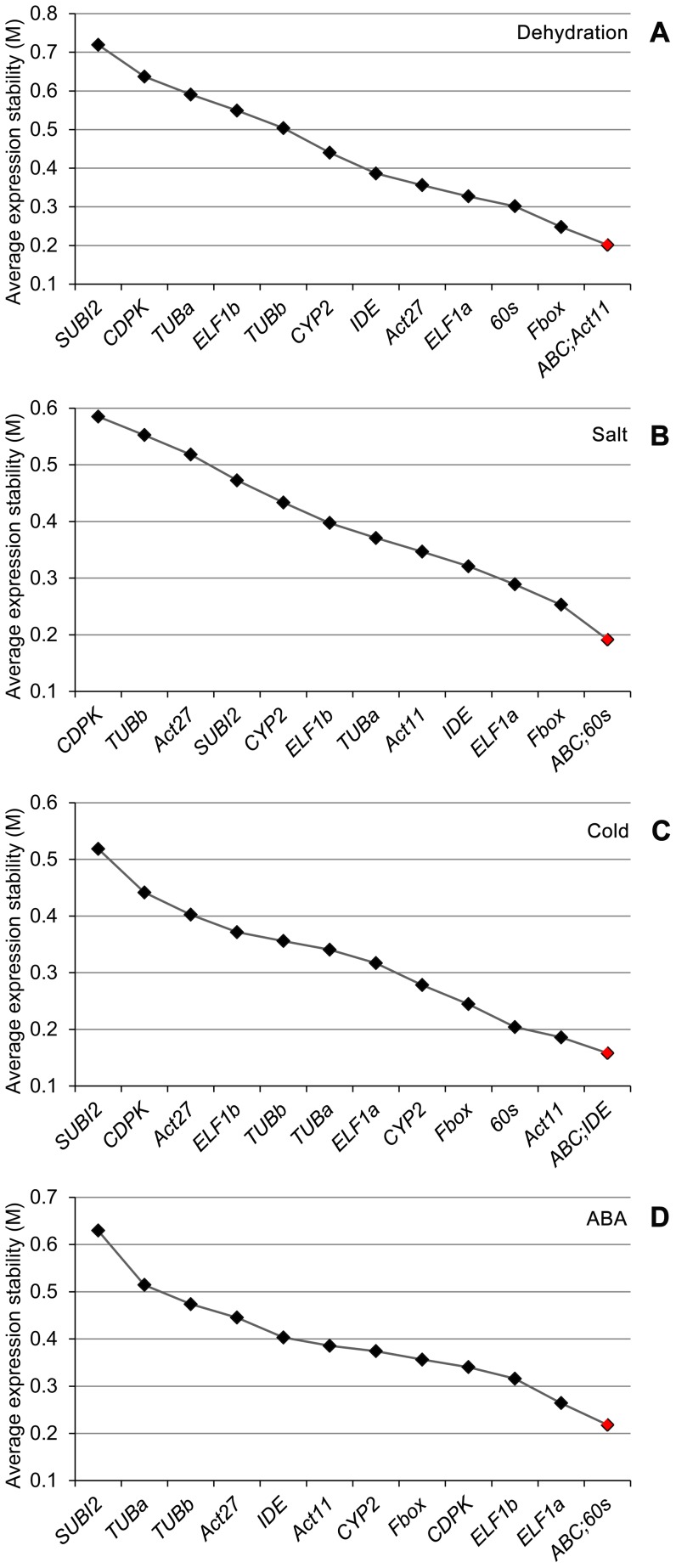
Expression stability and ranking for the candidate reference genes as determined by geNorm in both root and shoot tissues under individual stress or hormonal treatment. (A) Dehydration treatment. (B) Salt stress treatment. (C) Cold stress treatment. (D) ABA treatment.

Since a reference gene which is the most stable in one stress may be highly variable under other stresses, thus we analyzed the data based on individual stresses to search for the most stable reference gene(s) for each stress treatment. Under dehydration stress, results obtained from delta CT analysis (Table 3) revealed the top five most stably expressed genes for roots and shoots as *Fbox*>*ABC*>*Act11*>*TUBb*>*Act27* and *Fbox*>*60s*>*ELF1b*>*Act11*>*IDE*, respectively. The top five genes that can perform best in both root and shoot tissues are *Fbox*>*60s*>*IDE*>*Act11*>*ABC*. We then compared the data with that obtained by geNorm. In the roots, the gene encoding Fbox, ranked first by delta CT method, was not among the top five genes identified by geNorm which are *Act11*/*Act27*>*ABC*>*TUBb*>*TUBa* (Figure 2A). In the shoots, under dehydration stress *Fbox* was also ranked first by delta CT analysis; however, geNorm ranked *60s*/*ELF1b* as the best reference pair while *Fbox* was noted as the third best reference gene only (Figure 3A). The top five genes that perform best in both dehydrated roots and shoots determined by geNorm are *ABC/Act11>Fbox>60s>ELF1a* (Figure 4A).

**Table 3:** Average of standard deviations of delta CT obtained from tissues under individual stress.

	Dehydration			Salt			Cold			ABA		
	Roots	Shoots	R & S	Roots	Shoots	R & S	Roots	Shoots	R & S	Roots	Shoots	R &S
*60s*	0.2902	***0.4857***	***0.5346***	**0.3015**	**0.3537**	***0.3579***	***0.2196***	**0.2579**	***0.2778***	***0.3653***	0.3420	***0.3996***
*ABC*	***0.2145***	0.6269	**0.6452**	**0.2896**	0.4840	**0.4180**	**0.2441**	**0.2647**	**0.3557**	**0.4050**	**0.3278**	**0.4318**
*Act27*	**0.2179**	0.6418	0.7222	0.3128	0.7563	0.5739	**0.2424**	***0.2564***	0.4209	0.5245	0.5056	0.5560
*Act11*	**0.2159**	**0.5537**	**0.6363**	0.3823	**0.4164**	**0.4384**	0.2726	0.3457	0.3707	0.4838	**0.3282**	0.4563
*CDPK*	0.2437	0.9915	0.7685	0.6666	0.7199	0.6857	0.5454	0.5116	0.5909	0.5288	**0.3292**	0.4780
*CYP2*	0.2807	0.6573	0.8326	0.3622	**0.3792**	0.5192	0.2449	0.3092	0.3632	**0.4226**	0.3485	0.4756
*ELF1a*	0.2362	0.6186	0.8439	0.3238	0.4713	0.4462	0.2968	0.3152	0.4136	**0.4192**	0.3944	**0.4537**
*ELF1b*	0.2378	**0.5209**	0.6804	***0.2656***	***0.3277***	0.4703	0.2448	**0.2706**	**0.3563**	***0.3987***	***0.2959***	***0.4073***
*Fbox*	***0.2026***	***0.4538***	***0.4943***	0.3489	***0.3268***	***0.3707***	**0.2404**	***0.2382***	***0.3120***	0.4435	***0.3221***	**0.4320**
*IDE*	0.2439	**0.5967**	**0.5913**	***0.2743***	0.4947	**0.4441**	***0.2180***	0.2991	**0.3497**	0.4787	0.3714	0.5126
*SUBI2*	0.5441	1.2082	1.1174	0.5667	0.6558	0.6179	0.2898	0.3859	0.8414	0.9597	0.4756	1.1887
*TUBa*	0.2268	1.0299	0.8549	**0.2998**	0.5392	0.4750	0.2779	0.3671	0.3670	0.5854	0.3893	0.6987
*TUBb*	**0.2176**	0.8387	0.7954	0.3212	0.7498	0.5985	0.2936	0.3724	0.3851	0.5330	0.3625	0.5416

Data obtained for the top five genes are shown in bold letters, while those for the top two genes are in italic and bold letters.

### Evaluation of reference gene stability in soybean root and shoot tissues under salt stress

Unlike dehydration stress under which *Fbox* was identified as the best reference gene for roots, under salt stress *Fbox* was not among the top five genes for root tissues as determined by delta CT analysis (Table 3). Instead, *ELF1b* and *IDE* were found to be the best reference gene pair among the top five genes (*ELF1b*>*IDE*>*ABC*>*TUBa*>*60s*). In the salt stress-treated shoots, the top five genes are *Fbox*>*ELF1b*>*60s*>*CYP2*>*Act11*. With the exception of the *CYP2*, the remaining genes were also ranked among top five genes in dehydration-treated shoots (Table 3). Although, *ELF1b* and *Fbox* were ranked first in the roots and shoots, respectively, when the data for roots and shoots were combined and analyzed, *60s* was found to be the most stably expressed gene followed by *Fbox* (Table 3). When the data from the salt stress-treated roots were analyzed by geNorm, it turned out to be that the top three genes ranked by geNorm (*Act27*, *TUBb* and *ELF1a*) were not among the top five genes short-listed by delta CT analysis (Figure 2B, Table 3). In shoots, geNorm analysis indicated that except *ELF1a*, which was ranked first by geNorm but was out of the top five genes determined by delta CT analysis, the remaining top four genes, *60s*>*Fbox*>*ELF1b*>*Act11* were among the top five most stably expressed genes determined by delta CT analysis (Figure 3B, Table 3). The top two genes identified by delta CT method for both salt-treated roots and shoots (*60s* and *Fbox*, Table 3) were also among top three genes determined by geNorm (Figure 4B).

### Evaluation of reference gene stability in soybean root and shoot tissues under cold stress

Soybean adaptive responses to cold stress have also attracted a great deal of attention [51], [60], [61]. Thus, we next searched for the best reference genes to be used for expression analysis under cold stress among the 13 selected candidate genes. Using delta CT analysis, the top five genes that are most stably expressed in the roots and shoots of soybean seedlings under cold stress were *IDE*>*60s*>*Fbox*>*Act27*>*ABC* and *Fbox*>*Act27*>*60s*>*ABC*>*ELF1b*, respectively (Table 3). Four of the best performed reference genes ranked by delta CT analysis in each tissue were also shown to be the most stable genes by geNorm. Specifically, geNorm analysis demonstrated that *ABC*/*Act27*>*CYP2*>*IDE*>*60s* and *IDE*/*Fbox*>*Act27*>*ABC*>*60s* are the top five best reference genes for roots and shoots, respectively, under cold stress (Figures 2C and 3C). Although the *60s* gene was not the most stably expressed gene in any given tissues; however, it was the top-ranked gene to be used as a reference gene for cold stress across root and shoot tissues. The top five best candidate reference genes common for both tissues in cold stress were identified by delta CT analysis and geNorm are *60s*>*Fbox*>*IDE*>*ABC*>*ELF1b* (Table 3) and *ABC/IDE>Act11>60s>Fbox* (Figure 4C), respectively.

### Evaluation of reference gene stability in soybean root and shoot tissues under ABA treatment

ABA has been established as a key hormone involved in the regulation of plant responses to various abiotic stresses, such as drought and high salinity. Under these stresses, endogenous ABA level is increased, leading to up-regulation of many stress-responsive genes in ABA-dependent manner [62]. Thus, ABA treatment is often included in expression studies together with other stress treatments to determine whether the change in gene expression in response to stresses is ABA-dependent or ABA-independent [39], [51], [52]. Hence, a good reference gene for expression studies of stress-responsive genes under abiotic stresses should also stably express under ABA treatment. This prompted us to rank the performance of the selected 13 candidate reference genes to obtain the most stably expressed genes in roots and shoots that had been subjected to exogenous ABA treatment. The five top-listed genes in the roots and shoots under ABA treatment as revealed by delta CT analysis were *60s*>*ELF1b*>*ABC*>*ELF1a*>*CYP2* and *ELF1b*>*Fbox*>*ABC*>*Act11*>*CDPK*, respectively (Table 3). Only two genes, namely the *ABC* and *ELF1b*, were overlapping in the lists of the top five most stably expressed genes in each tissue, suggesting that the most stable reference genes are not only stress/hormone-dependent but also tissue-dependent. When geNorm was use to analyze the expression stability, we found that all the top five genes listed for the roots (*ABC*/*60s*>*ELF1a*>*ELF1b*>*CYP2*) were also ranked as the top five by a geNorm analysis although the order was different (Figure 2D, Table 3). As for the shoots, with the exception of the *ABC*, the other four top-listed genes determined by delta CT analysis were found among the top five genes as suggested by a geNorm analysis (*Act11*/*Fbox*>*ELF1b*>*IDE*>*CDPK*) (Figure 3D, Table 3). In addition, we also searched for reference genes that can be used to compare gene expression across tissues under ABA treatment. The data for roots and shoots were combined and analyzed using the delta CT approach. Results on Table 3 demonstrated that the top five most stably expressed genes in both roots and shoots of soybean seedlings were *60s*>*ELF1b*>*ABC*>*Fbox*>*ELF1a*. The same five genes were also identified by geNorm, although the exact order was different (Figure 4D).

### Validation of the usefulness of the reference genes identified from this study

Next, to validate the performance of the reference genes identified in this study on known abiotic-stress inducible genes, we quantified the expression of four *GmNAC* genes, which were reported to be up-regulated by dehydration [39], [41], and normalized their expression levels using a representative least stable reference gene (*SUBI2*) and two representative good reference genes (*Fbox* and *60s*) (Table 3). As shown in Table 4, with a fold-change threshold of 2.0, we would have consistently failed to detect the induced expression in the roots and shoots for *GmNAC019* and in the roots for *GmNAC043* and *GmNAC092*, if *SUBI2 -* a least stable reference gene under dehydration stress - were used as a reference gene. However, the up-regulation of these genes was reliably detected when the expression levels were normalized with the most stable reference genes, such as *Fbox* or *60s*. When the induction level was high, as that of *GmNAC85* in both tissues or that of other *GmNAC* genes in the shoots, normalizing to bad reference genes, such as *SUBI2*, could still be able to detect the induction but was shown to underestimate the induction level by 3- to 4-fold (Table 4).

**Table 4:** Differential expression of known dehydration-inducible genes normalized with *60S*, *Fbox* or *SUBI2* reference genes.

		*GmNAC19*					
		Fold Changes			Standard errors		
		*SUBI2*	*60s*	*Fbox*	*SUBI2*	*60s*	*Fbox*
0 h	Root	1.00	1.00	1.00	0.14	0.27	0.22
Dry 2 h		1.26	2.49	2.58	0.10	0.27	0.27
Dry 10 h		1.78	2.32	3.22	0.18	0.05	0.14
0 h	Shoot	1.00	1.00	1.00	0.11	0.04	0.04
Dry 2 h		0.96	3.13	3.75	0.15	0.09	0.06
Dry 10 h		1.12	18.66	13.93	0.09	0.05	0.04
		***GmNAC43***					
0 h	Root	1.00	1.00	1.00	0.24	0.23	0.19
Dry 2 h		1.50	3.06	3.18	0.22	0.23	0.23
Dry 10 h		1.68	3.06	4.24	0.11	0.04	0.14
0 h	Shoot	1.00	1.00	1.00	0.04	0.05	0.05
Dry 2 h		17.26	25.42	30.51	0.16	0.12	0.08
Dry 10 h		25.69	101.32	75.61	0.04	0.09	0.08
		***GmNAC85***					
0 h	Root	1.00	1.00	1.00	0.24	0.23	0.18
Dry 2 h		3.06	6.23	6.46	0.16	0.18	0.16
Dry 10 h		4.96	9.02	12.50	0.24	0.15	0.24
0 h	Shoot	1.00	1.00	1.00	0.05	0.02	0.02
Dry 2 h		58.75	86.51	103.83	0.16	0.13	0.07
Dry 10 h		139.24	549.09	409.76	0.02	0.08	0.06
		***GmNAC92***					
0 h	Root	1.00	1.00	1.00	0.29	0.27	0.22
Dry 2 h		1.22	2.49	2.58	0.27	0.27	0.27
Dry 10 h		1.28	2.32	3.22	0.12	0.05	0.14
0 h	Shoot	1.00	1.00	1.00	0.02	0.04	0.04
Dry 2 h		2.12	3.13	3.75	0.13	0.09	0.06
Dry 10 h		4.73	18.66	13.93	0.02	0.05	0.04

### Conclusions

Upon exposure to stresses, soybean plants activate numerous signaling pathways to respond to the adverse environmental stimuli [1]–[3]. The first step toward adapting to stresses is to induce or repress the expression of various genes. To accurately understand the mechanisms regulating stress responses at transcriptional level and to identify the appropriate stress-responsive genes, stably expressed reference genes are needed if qRT-PCR is used for expression profiling. Before this study was conducted, there have been no comprehensive reports on the search for reference genes for soybean under various abiotic stress conditions, except a study which surveyed for reference genes for expression study under drought stress in soybean. However, this study used only four candidate genes, namely the *Gmβ-actin*, *GmGADPH*, *GmLectin* and *GmRNA18s*, among which the *Gmβ-actin* and *GmRNA18s* were found to be the best reference genes for soybean under their experimental conditions [47]. This prompted us to choose 13 candidate reference genes from literature and ranked their performance as the best reference genes under various abiotic stress and hormonal treatments, including dehydration, salt, cold and ABA treatments. Because most of the recent studies showed the preferable use of root and shoot tissues of soybean seedlings for gene expression profiling under abiotic stresses using qRT-PCR [39]–[41], [55]–[58]; we tested the expression stability of the 13 selected candidate genes in these tissues of 12-day old soybean plants. Our results revealed that there is no single reference gene that can be best for all conditions and/or both the tissues. Instead, we found that the best reference genes are tissue- and/or stress-dependent. In addition, although there is high agreement in overall among the top five genes ranked by either delta CT analysis or geNorm, the best reference genes inferred by one method may not be exactly the same ones as determined by another approach; a phenomenon which has also been observed earlier [63], [64]. As geNorm has been known to rank co-regulated genes as the best reference genes, our conclusions were made based primarily on the result of delta CT analysis. The result obtained by geNorm is provided to readers as additional information source. The most stably expressed reference genes identified in this study were shown to help detect subtle differential rates of gene expression as well as avoid the underestimation of the induction/repression levels.

Taken together, using delta CT analysis with consideration of geNorm result, we suggest that the following gene pairs (summarized in Table 5) are suitable for use as reference genes in the respective tissues and under specific stress:

- For dehydration, *Fbox*/*ABC* should be used for the roots and *Fbox*/*60s* for the shoots, respectively. The *Fbox*/*60s* gene pair is also the best for comparing expression between roots and shoots under normal and dehydration conditions.
- For salt stress, *ELF1b*/*IDE* and *Fbox*/*ELF1b* should be used as reference genes for roots and shoots, respectively. For reference in both root and shoot tissues under salt stress, *60s*/*Fbox* is the best gene pair.
- For studying expression change by cold stress individually in the roots or the shoots *IDE*/*60s* and *Fbox*/*Act27* are the best reference pairs, respectively. For analysis gene expression under cold stress across root and shoot tissues, *60s*/*Fbox* should be used.
- To examine gene expression under ABA stress in roots, shoots or across these two tissues, *60s*/*ELF1b*, *ELF1b*/*Fbox* and *60s*/*ELF1b* are the best reference genes, respectively.
- Based on the results of this study, when comparison of the expression profiles of a gene in response to various stresses is of interest, the *ELF1b*/*60s*, *60s*/*Fbox* and *60s*/*Fbox* reference gene pairs are recommended for roots, shoots and both tissues, respectively. When a single reference gene is required, *60s* should be the best choice.

**Table 5:** Recommended reference genes for qRT-PCR of soybean tissues under various abiotic stresses.

	Roots		Shoots		Roots & Shoots	
**Dehydration**	*Fbox/ABC*	*ELF1b/60s*	*Fbox/60s*	*60s/Fbox*	*Fbox/60s*	*60s/Fbox*
**Salt**	*ELF1b/IDE*		*Fbox/ELF1b*		*60s/Fbox*	
**Cold**	*IDE/60s*		*Fbox/Act27*		*60s/Fbox*	
**ABA**	*60s/ELF1b*		*ELF1b/Fbox*		*60s/ELF1b*	

## Supporting Information

Figure S1 **Melting curves of the amplicons.** The amplicons were produced by the primer pairs used to quantify the expression stability of the 13 candidate reference genes.
(TIF)

Table S1 **Raw data of delta CT analysis of the expression stability of the candidate reference genes.**
(XLS)

Dataset S1 **Primer locations on respective transcript.** Primer sequences are in orange or in red boxes. Primer pairs with positions on two differently colored portions of the sequence are on two different exon or UTRs.
(PDF)
