# Correction: Evaluation of Candidate Reference Genes for Normalization of Quantitative RT-PCR in Soybean Tissues under Various Abiotic Stress Conditions

**DOI:** 10.1371/annotation/6a5108f5-50f8-418e-854d-8f3eb94e6fc0

**Published:** 2012-10-03

**Authors:** Dung Tien Le, Donavan L. Aldrich, Babu Valliyodan, Yasuko Watanabe, Chien Van Ha, Rie Nishiyama, Satish K. Guttikonda, Truyen N. Quach, Juan J. Gutierrez-Gonzalez, Lam-Son Phan Tran, Henry T. Nguyen

There were errors in the Funding statement. 

The correct statement is: This work was supported in parts by the National Center for Soybean Biotechnology and the Missouri Soybean Merchandising Council to HTN and by a grant (No. AP24-1-0076) from the RIKEN Strategic Research Program for R & D to L-SPT. DTL was supported by RIKEN Foreign Postdoctoral Fellowship. The funders had no role in study design, data collection and analysis, decision to publish, or preparation of the manuscript. 

