# Supplementary figures and images for: Evaluation of Candidate Reference Genes for Normalization of Quantitative RT-PCR in Soybean Tissues under Various Abiotic Stress Conditions
Source: PLoS One. 2012 Sep 28;7(9):e46487. doi: 10.1371/journal.pone.0046487 (PMC3460875; doi:10.1371/journal.pone.0046487)

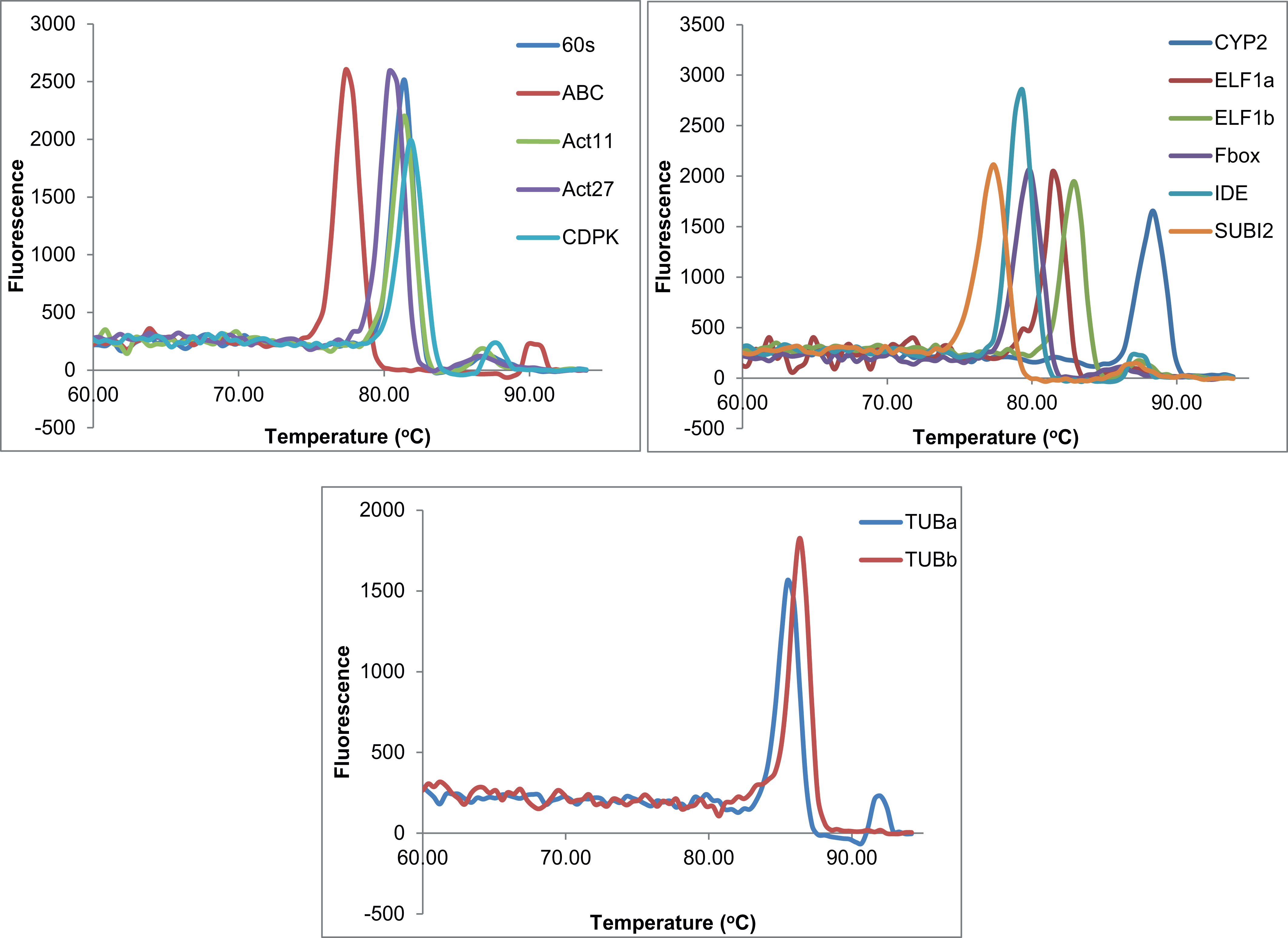

Supplement: Figure S1 — Melting curves of the amplicons. The amplicons were produced by the primer pairs used to quantify the expression stability of the 13 candidate reference genes. (TIF) [file pone.0046487.s001.tif]
