# Supplementary material for: Evaluation of Candidate Reference Genes for Normalization of Quantitative RT-PCR in Soybean Tissues under Various Abiotic Stress Conditions
Source: PLoS One. 2012 Sep 28;7(9):e46487. doi: 10.1371/journal.pone.0046487 (PMC3460875; doi:10.1371/journal.pone.0046487)
Supplement: Dataset S1 — Primer locations on respective transcript. Primer sequences are in orange or in red boxes. Primer pairs with positions on two differently colored portions of the sequence are on two different exon or UTRs. (PDF) [file pone.0046487.s003.pdf]

Le *et al.*, Evaluation of candidate reference genes for normalization of quantitative RT-PCR in soybean tissues under various abiotic stress conditions

Dataset 1. *Primer locations on respective transcript. Primer sequences are in orange or in red boxes. Primer pairs with positions on two differently colored portions of the sequence are on two different exon or UTRs.*

```
>Glyma17g05270.1
AGGGCTTTTTCAGTTTTCCTCTTCTGAATCCACCGCCACCGCAGCGTGGACGGAATCAGAGTGCAGCCATAGCAGCAGCAGCAGCAGTTATTGAAGTGC
TGCTCTAGTTTGAGGCACAATGAGATTGGAGAAATGCTGGTTTTGCTCTTCAACCGTATACCCCGGACATGGAATCCAGTTTGTTCGTAATGATGCAAAG
ATTTTTCGGTTTTGTAGATCAAAATGCCACAAGAACTTTAAAATGAAGAGAAATCCTCGTAAGGTAAAGTGGACCAAGGCATATCGTCGAGTGCATGGAA
AGGATATGACTCAGGACTCAACCTTTGAGTTTGAGAGAAAACGAAACAGGCCTGAAAGATATGACAGGAATCTTGCGGAGAATGTCCTGAAGGCCATTCC
TAAGATTGATAAAATCAGAGTCACCAGGGAGGAGAGACACCATAAGAACAGGATGAAAGGAAAAAAGGAAAAGCTGCTGAAGGAGGCAGTGAAGGAGTTG
GAGCAGGGCATCAGTTTGGTCAAAGCTCCTTCTGTGCTTCAACAGGATCCATCTCTCACATTACCAAAGATCAAAGTCAAGGTTTCCCAACAGCAATCAG
AGGAGAATCATGCCATGGAAGAGTAATTCTAACAATGCTGCACTTAATTTTTGCCGTTTTAATATTTGTTGCTAAATTAGTTGCCTGTAAGGTCCAGTTT
ACCAGTTTCCTTGTGATCATTAATGTGAATGATGTGTTCTTCAAACATTCGATGTATTTGTCTCTTAAAACTATGGTTCTCTGAGATAGTCAACTATTAT
AAAGGAAATCGGAAAGTTGTTGCTATAGCCT
```

60s; 60s Ribosomal protein L30

```
>Glyma12g02310.1
AAAATTTTGGGCAGCATAGATCTGAATGGAGAGCGAGGTGGTGTCCATTGCAGTTTTCTAATGAAAACAACAAAATAGACACCTGTTAAGAAGGAGGACA
ACGATGGTGCTGCCGCTATTTACTGCTTTCATTCTCTTTGCTTTTATTCCGAATGGATTGTTAGCAGTTCCTTCCACCGTCCCGGCATTCCTTTGGTCAT
CACATTATGAACTGGCCTCTGAAAATATATTGAAGGAATCTGTAAATTACCAAGTCATTTCTCCGAAGGATCTAGCAAAATCTATTTTATCTGAAGCAGG
CTGGTCAAATTTTCTGTGCAAAGGAAAGAAATTTCATGAGCCTCTGGATCTGGCACTCTTATTCGTTGGTAGAGAGTTACAATCTTCAGATTTAAGCATG
AACAAACATGCAAACTCAGCTCTTTTGGACCTGCTCAAGAACTCTTTCGCCAGATCCAACACTTCTGTGGCATTTCCCTATGTTTCTACATCAGAGGATG
TGCTTTTGGAAAACTCGTTGGTTTCAGGATTTGCTGAAGCCTGTGGAGATATGGGAATTGGCAATGTTGCTTTCCATGGATCTTGCTCCATTGATGATGC
AAATCATGAAGAAATCACAACTTTGCACTCAGTTCAAGACTATTTGACCAAGAGGATGGAAGAGAGTCACAGAGGGAAAACTGATTTGGTTGTGTTCTGC
AATGAAGGCTCTCAAGCTCTTGAGAATGTTGACAGGACACAATCTGAAGGGGAAGTTTTATCCAATCTTATCAATTCTGTGGAGGAATCTGGTGCAAAAT
ATGCCATTCTTTATGTGTCAGATCCCTCAAGGTCAATCCAGTATCCTTCTTACAGGGATCTGCAAAGGTTTCTAGCAGAAAGTACAGCCGGGAATGAATC
AACCAATTCCACAATTTGTGATGAAGTCTGCCAGATTAAATCATCTCTTCTAGAGGGAATTTTAGTGGTAAATTTCCTTGTCCATTTTTACAAGTGCAGT
AGCTCGATGACCTGGTATAGTTTTGCTAATAATTTTGATATCGGGCCTTTGCTGCATGATGGGAATTGACACCCCAACAAGATTTGAGACGCCACAAGAG
TAATGATCCATTTGCTGAAGCTAATAAATGTTGTGATGCTCTCGGTCTACTGGATTAAAATATTACTGCTATATTATTTGCGAATGTTACGGAGGCTCTG
ATAAGTGTTCTGTAGTTGATGACTTATCTCAGGGACTTGACATATTGAATAATAAAATAATTTGTATCAGTATATAAAAATGAAACTTCAAGCTTAGGTT
TAATTTTGATCCCGCCACCATTCAGATTATGTAAAATTTGAAGAAATTTCAGATTATGTGTTGTATTTTTTTAACTGTTCGCAAATTTGCCCCATTCGTT
GTGCATAATTGCTGATCGATGCAGTTGCAAGTTTATTTCTGCTGGGTTTTGAATTGAAGTTTGAAGTTGATTTGAAGAATGTTTAGCAATATGGTAAAAT
TTCGAGTTAAAGTTTGAGATTGAAGTTAAAGATATTAACAAGTGGAAGTTCTAAAAGTAGACGAGTGTCAGATGAAGGATTTCAAGGTAAGGGTATAAAT
TGAGAGGAAGTTAATAACAATTACCTTCGCTGCATGCATTGAGTGAGATGCTGAAATTTTATGCAATTTGTTTACACGTGTTTGTATAGCATATATCAAT
CGTTATGGTTGGAAATCTGAATAGGATATTTATATTCTAATTTTTATTGTTGAAACAGATCGAATAGTGTTATGTTCTATACATAAATAAATGGTTACTG
GTTTAACAAGTT
```

ABC, ATP-binding cassette transporter

ATGGCAGACGCTGAGGATATTCAACCTCTTGTTTGTGACAATGGAACAGGAATGGTTAAGGTACATGATGGATCTTTTGTTTTTGTATTATTTGTTGAAT
AAACTTACTGTCATACTCTTGTTGGTTTTATGGGAATATCATTTTACATTAATGTTACTTTTGTGAATTTGAAAAATTAATTGCAACAAATATTTGTAGG
CTGGTTTTGCTGGAGATGATGCCCCACGTGCTGTGTTTCCCAGCATTGTAGGTCGTCCTCGTCACACTGGTGTGATGGTTGGCATGGGCCAAAAAGATGC
ATATGTTGGTGATGAGGCACAGTCTAAGAGGGGTATTTTGACTCTCAAGTACCCCATTGAACACGGAATTGTGAGCAATTGGGATGATATGGAAAAGATT
TGGCATCATACCTTCTATAATGAGCTTCGTGTTGCCCCAGAAGAGCATCCAGTTCTCTTAACTGAGGCCCCTCTTAACCCTAAGGCTAATCGTGAGAAGA
TGACCCAAATCATGTTTGAGACCTTTAACACCCCTGCTATGTATGTAGCCATCCAGGCTGTTCTTTCACTTTATGCAAGTGGTCGTACAACTGGTAAGCC
ATTTTTTATTTGTTTGATTAACTTGAGGTTGTATAGTATGAACTTATCTGGGAACTTTGAAATTGAGATAAGCATATGTCAACATCCATGAGAATCTATA
AAGTCTGTATGTTCAACATTGCATTTTTGGATATTTTAAAATAAACTGAAAATGATTCATTTCATTGTGAATGATTTCACTTGACATATTACTAAATGGA
GAAAAAACAAATTCTGTGAACTGGGAATTATGATTGTAGAAAACTGCTGTAAAGATTCCTTATATCATGTGATCGACTTTTAAAACTTTATTTATTTGCC
TTCTCTTTGCAGGTATTGTGTTGGATTCTGGTGATGGTGTCAGCCACACTGTCCCTATCTATGAGGGTTATGCCCTCCCACATGCCATCCTTCGTCTTGA
TCTGGCTGGTCGTGACCTCACAGATTTCTTGATGAAAATCTTGACTGAGCGTGGTTATTCCTTCACCACCTCAGCCGAGCGGGAAATTGTAAGGGATGTG
AAAGAAAAGCTTTCTTACATTGCCCTTGACTACGAGCAAGAACTGGAGACAGCCAGGACCAGCTCGTCTGTGGAGAAGAGCTATGAGTTGCCTGATGGGC
AGGTCATCACCATTGGCGATGAGCGTTTCAGATGTCCAGAGGTTCTGTTCCAACCATCCATGATTGGAATGGAAGCTTCAGGCATTCACGAGACAACATA
CAACTCCATAATGAAGTGCGATGTTGATATCAGGAAGGACCTCTATGGTAACATTGTTCTTTCAGGAGGAACAACCATGTTCCCTGGTATTGCTGACAGA
ATGAGCAAGGAAATTTCGGCATTGGCACCCAGCAGCATGAAGATCAAGGTTGTAGCACCACCGGAGAGAAAATACAGTGTCTGGATCGGTGGTTCTATCT
TGGCATCTCTTAGCACCTTCCAGCAGGTTTGTTCTCTTCCTTTATTCTTTTTCCTGCTTCTTGGAATGTTATGTTGTTGTGAGATAGTACATTCTTCTAT
GATAATAATGACACACTTTTGTTTTGAGTGCAGATGTGGATTGCAAAGGCAGAATATGACGAGTCTGGTCCATCAATTGTGCACAGAAAGTGCTTCTAAA
ACTGTTTGAGAGATAGGGTTATTGAAGCGAAAGACTACTATCATCATGATTTGTCTGGAAGCAATGCATTGCAGATTGTAACCCTTTCATTCTAGCTTT

Act11; Actin, reversed primer located after the current annotated version of the transcript but these primers were used in *BMC Mol. Biol. 2008, 9:59,* white background sequences are intron in Glyma database

```
>Glyma19g32990.1
ACACACACAAATTCACACAAACATAAAAAAAAAAAAAAAAAACACACACACTCTCATACACACGTTGTCGCGCACACATTCCTTCATTCCGCAGCAAC
AAACAAACATCTTTTACCTTAAACAACAATGGCCGATGCCGAGGATATTCAACCCCTCGTTTGCGATAATGGAACCGGAATGGTCAAGGCTGGTTTTGCT
GGAGATGATGCACCGAGGGCTGTGTTCCCTAGCATTGTGGGGCGTCCACGTCACACTGGGGTGATGGTTGGGATGGGGCAGAAGGATGCGTATGTTGGGG
ACGAGGCTCAATCCAAGAGGGGTATTTTGACTCTCAAATACCCAATTGAGCATGGAATTGTGAGCAATTGGGACGACATGGAGAAGATCTGGCATCACAC
TTTCTACAACGAGCTTCGTGTGGCTCCTGAGGAACACCCTGTGCTTCTCACCGAGGCACCTCTTAATCCTAAGGCTAATCGTGAGAAAATGACTCAGATC
ATGTTTGAGACCTTCAACACCCCTGCTATGTATGTCGCTATCCAGGCCGTGCTTTCCCTTTATGCTAGTGGCCGTACAACTGGTATTGTTCTGGACTCTG
GAGATGGTGTCAGTCACACGGTTCCTATCTACGAAGGTTATGCCCTCCCACATGCAATCCTGCGTTTGGACCTTGCAGGGCGTGATCTCACTGATGCCCT
CATGAAAATCTTGACTGAGCGTGGTTACACTTTCACCACATCTGCGGAACGGGAAATTGTGAGGGACATGAAGGAGAAACTGGCCTACATTGCTCTGGAT
TATGAGCAGGAGTTGGAAACTGCCAAGACCAGTTCAGCTGTTGAAAAGAGCTATGAGCTACCTGATGGGCAGGTGATCACGATTGGCGCTGAACGATTCC
GATGCCCTGAAGTTCTGTTCCAGCCATCCATGATTGGGATGGAATCTCCTGGTATCCATGAGACAACATATAACTCTATCATGAAGTGTGATGTCGACAT
TAGGAAGGATCTCTATGGTAACATTGTCTTGAGTGGTGGTTCCACAATGTTCCCTGGCATTGCTGATAGGATGAGCAAGGAGATTACAGCATTGGCACCA
AGTAGCATGAAAATTAAGGTTGTAGCACCACCAGAGAGGAAGTACAGTGTCTGGATTGGAGGCTCCATCTTGGCTTCCCTCAGCACCTTCCAACAGATGT
GGATTGCGAAGGCAGAGTATGATGAATCTGGACCATCAATCGTACACAGGAAATGCTTCTAAGTTATAATCATGGAGTGTGAAAGCTGGACCAGGGAAAT
TACTATTTATACAAATACTACAAAAATACCATCTAGTGGTTGAGGAACTTTCATTTCCTACTCTTTACCATCCTTTTATCTATCTTGTTTTTGTGTTTTC
CTTTCTTTGGTATGTTGAGATAAGAGCATGAAGGCTAGCAAGATATGTAAGATTCTTTTTTTTTTCTCCCGTTCTGTTGTAGAAGAGATGTGAATTGTTA
CCTATTTGGTTTGGTTGAATTAACAAATTCTTTTCATGAAGTATTCTGATTAACATAGTGGTACGGTACGTGCTTGATTTA
```

Act27, Actin

```
>Glyma12g02790.1
CAACCCCAAGCAAAACCCTAATCCCTCTTTCTTCCTCTTCCTCAGTAGTGCGATTTTCGATTCTCTTCTCTGCAACTATGCCTAACCCTAAGGTCTTCTT
CGACATGACCATCGGCGGTCAACCTGCCGGCCGCATCGTGATGGAGCTCTACGCCGACGTGACTCCGAGCACCGCCGAGAACTTCCGCGCGCTCTGCACC
GGCGAGAAGGGCGCCGGGCGGAGCGGCAAGCCCCTCCACTACAAAGGCTCCTCCTTCCACCGCGTGATCCCGAACTTCATGTGCCAGGGCGGCGACTTCA
CCGCCGGAAACGGCACCGGAGGCGAGTCGATCTACGGCGCCAAGTTCGCCGACGAGAACTTCGTGAAGAAGCACACTGGTCCCGGCATCCTCTCCATGGC
GAACGCCGGTCCCGGAACCAACGGATCTCAGTTCTTCATCTGCACGACGAAGACGGAGTGGCTCGACGGAAAGCACGTCGTGTTCGGACAGGTCGTCGAG
GGGATGGACGTCGTCAAGGAGATAGAGAAGGTCGGATCCAGCTCCGGCAGGACCGCCAAGCCTGTCGTCGTCGCCGACTGTGGTCAACTCTCTTAGATAG
ACGACGTTGACCCTAGGCTTTTATCTCTGTGTCGGTGGCTCTGAATGTGTCGTTTCTTTCGGTGTCGTTTTTGCCTTTTTTTTCTCTTTCCCATGTCGTT
TAGGGGATTGCGGTGTTATGGATATCATTTCGAAACCCTTCCCCTTTGAATCCTTCCTTTCCTTTCTAAAATATCAGTATCATGTGGTTCGTTCATAGAG
TGAATGGAGTCTGTTATGAGAAACATAAAAAAATATGATGAGCTACCTTCAATAAAAATTAGGACCTCTATTATTCGCCTAGTAATTCTCTTTAATCTTC
GATTATTTTTTTATATACGTTTAGTTGTTTCTGATTATAAATTGTGGAAAATTGGCGTTTATTAAATACCATAGTTTACGATAATTAGAACCTACAACT
AGGAACGTTCATTGGTTTGATGGATTGATATTTGTGCAGATAATACATACGACTGTGTTATTTTTGATG
```

CYP2, Cyclophilin

>Glyma19g07240.1

```
ATCACTCTGCTGCCTCGCTCTGTTTCTGTCTCTGTGTTTGCGGCTGAGGATTCCGAACGAGCGACCTTCTTCGTTTCTCGCAAAGTTTAAGTCATCATGG
GTAAGGAAAAGACTCACATCAACATTGTCGTCATTGGACATGTCGACTCTGGGAAGTCAACTACCACTGGTCATTTGATCTACAAGCTTGGAGGTATTGA
CAAGCGTGTGATTGAGAGGTTCGAGAAGGAGGCTGCTGAGATGAACAAGAGGTCATTCAAGTATGCCTGGGTGCTTGACAAGCTCAAGGCTGAGCGCGAA
AGAGGAATTACCATTGATATTGCTTTGTGGAAGTTTGAAACCACCAAGTACTACTGCACAGTCATCGATGCCCCGGACATCGTGACTTTATCAAGAACA
TGATTACTGGTACCTCCCAGGCTGATTGTGCTGTCCTTATCATTGACTCCACCACTGGTGGTTTTGAAGCTGGTATTTCTAAGGATGGACAGACTCGTGA
GCATGCTCTTCTTGCTTTCACCCTTGGTGTGAAGCAGATGATCTGCTGCTGTAACAAGATGGATGCCACTACCCCGAAGTACTCTAAGGCTAGGTATGAT
GAAATTGTGAAGGAAGTCTCTTCCTACTTGAAGAAGGTTGGTTACAACCCAGACAAGATTCCATTTGTGCCCATCTCTGGTTTCGAGGGTGACAACATGA
TTGAGAGGTCCACCAACCTTGACTGGTACAAGGGACCAACTCTCCTTGAGGCTCTTGACCAAATCAATGAGCCCAAGAGGCCCTCAGACAAGCCACTCAG
GCTTCCATTGCAGGATGTCTACAAAATTGGTGGTATTGGTACCGTGCCAGTGGGACGTGTTGAGACTGGTGTCGTGAAGCCCGGTATGGTGGTGACTTTT
GGTCCCACTGGGCTGACAACTGAGGTTAAGTCTGTTGAGATGCACCATGAGGCTCTCACAGAGGCTCTTCCAGGTGACAATGTTGGCTTTAATGTGAAGA
ATGTTGCAGTCAAGGATCTCAAGCGTGGTTTTGTTGCATCCAACTCCAAGGATGACCCTGCCAAGGAAGCTGCCAACTTCACATCTCAAGTCATTATCAT
GAACCACCCTGGCCAGATTGGAAATGGATACGCACCGGTGCTTGATTGCCACACTTCTCACATTGCTGTCAAGTTTTCTGAAATCTTGACCAAGATTGAC
AGGCGATCTGGTAAGGAGCTTGAGAAGGAGCCCAAATTTTTGAAGAATGGTGATGCAGGTATGGTTAAGATGATTCCAACCAAGCCCATGGTGGTTGAAA
CTTTCTCTGAGTATCCTCCCCTTGGTCGTTTTGCTGTGAGGGACATGCGTCAAACTGTGGCTGTCGGAGTCATCAAGAGTGTTGAGAAGAAAGACCCCAC
CGGAGCCAAGGTCACCAAGGCCGCTGCCAAGAAGAAGTGATTGCATTTGGGCAATTTTGCTAGCACATGTGATCATCATCGTGGTTACTCCTTTATAGTA
GTTTTATCCTTGCAGAGTCTTAGGTGTTTTGTTTAAGTTATATTTTTAAGTTTCTGCCGATTTCATGTAGCCGTAACTTTCAAAACTAGGTTCTTGATCG
GCGGTGGTCAATTTTCATTGCTGTTTGTTTTTGATGAGTACTGTTTTTTGTTTTGATGGTAAGAAGAGTCTGAGATATTTCGAATTTCACAAGCAGCTAT
AGGGTTTTAGTCCATTTCCTTTGCTGCTGAGGGATGTTTTAAGTTGCATTTAATTTATAACGAAGTTTTATAAACTGTTTATGGTTTAAAGGCTATTATT
CTTT
```

ELF1a; Eukaryotic elongation factor 1 alpha

>Glyma02g44460.1

```
GTGACTGTGCATGCTATAAATATCAATTATTGAGCATTGAAGGAGTCTCTCTTTTTCACTCACTCACTCTGCACTCACCACTGCCACACTTCAATCGC
ACTAATGGCTGTCACCTTCTCAGATCTACACACCGAAGAGGGCATCAAATCCCTCGATCAATTCCTTTCTGGGAAAACCTATATTTCTGGGGATCAGTTG
ACAAAGGATGATATTAAAGTGTATGCAGCTGTTGTTGAAAAGCCAGGGGACACTTTTCCTAATGCTGCCAAGTGGTACGATGCTGTCTCTTCTCAACTTG
CTCCAAGCTTTCCTGGCCACGCTCAAGGGGTAAGATTCAGTGGCGCTGCTGCTCCAGCTGAAGCTGCACCTGCCAAAGCGGCTGCCACTGCTGAAGAAGA
TGATGATGATCTTGATCTCTTTGGTGATGAGACAGAGGAAGATAAGAAGGCAGCAGAGGAAAGGGAGGCTGCTAAAAAGCCTGCCAAGAAAAAAGAGAGT
GGCAAGTCTTCTGTCCTTCTCGATGTTAAGCCTTGGGATGATGAGACCGACATGAAGAAGCTGGAAGAGACTGTCCGCAGTATTGAGATGCCTGGTTTAT
TGTGGGGAGCATCCAAACTGGTTCCTGTGGGATATGGGATCAAGAAGTTGCAGATCATGATGACTATTGTTGATGACCTTGTATCCGTGGACACCCTTGT
TGAGGAACGCTTGACAGTTGAGCCATGCAACGAGTATATCCAGAGCTGTGACATTGTTGCATTCAACAAAATTTAAGTTTTCTTGTTTATTTTGATTTTA
GCCATTTTCAAGCGGCTGATCAGTCTGTTACTCTTCCTTTCATTGTTCATGTTTGGTCTCTTTCAATAGTGGTTTTGTTGCAAAAATTGTGAGAAGTAAA
AGCAATATATTAGGGTGTGTTTGGTTTATATTTTTATTTTCTGTTTTTATTTTATTGTTTTCAGTTTTTGGTTAATTTGGGAAAATATTTTCATTGAAAA
TGAAAATAGAAATCCAACAAAATACATTTTCATTATCATTTTTTATTTTATATAAAAATGAAAATAGAAAATAATCAAATCAAACACCCCTTAAATATCA
TGTTATGGTGTATCTTTGAATATTATTTCTTGAATTAGATTTATGGATTAAGATAACTGAACGATGTGCTTCAATT
```

ELF1b, Eukaryotic elongation factor 1 beta

>Glyma12g05510.1

GTTTTTGTTGTTTGGTGTTATTTTATTTTTTTAATCCGTAAAAATAAAAAGAAATACAACATTTCACCCACCCTTGTTGTTATGTGAGTGCGGCAATAGT
GGAATAGCGGTAGAGTGTGCGATCGAGAAAGCAGAAAGCAGAAAGATGGGGTTGGAATCGGTGGGAGATTTAGCGATTAACGTGATTCTGAAAAAATTAG
GAGCCCAAGACATTGCGAGAGTGGCGTGTGTGAGCAAAAGGTTCAGTTCTTCCGCTTCCGATGACACTCTTTGGATCAATCTCTGCTTCAATGAACTCGC
TTTGACACAACCCCTCGATCATCTCGGAAACCCTCTCTCTTCCTTCAAGGAATGCTATCTAGCATGGAGAGGAGCTTTTGTTATGTACCCTTGGTCTCTT
GTTAAGCGTGTAAAAAGGTGCTGGGATAGAATAAAAACCTGGTTGACCAATAATTTTCCTGAAGCGGAGGCCACTCTTTGTAAAGGTGCAACTGAAGCTG
ACATTCAGGAGTTGGAGAATGTATTAAAGGTGAAATTGCCTCTTCCTACAAGGATCCTTTATCGCTTTCACAATGGGCAAGAATTTGCAAAGGCAGATCC
AGAAACTAGTACATTTGGCAGATCTTTGGGTCTAATTGGTGGCTACTCCTTCTATGGTCATTTGGTGAATGTTTATCTATTACCTATATGTCAGATAATC
CTAGAAACTCAGCAAACTAGGCGTCGCTTGAGCTTTTTAAGAAGATCAAAGTATGTTCTTGTGGCTGCTTCATCCACATACAGTAGAAAGTTGTTTTTCC
TCAACTGTACCAATGGTCAACTATATGTCGGGACCAGGTCTCCTCTTACCGAAAGAGACATAATCCCTTGTGTACCTCATGACCTGATTAGTTTACATCA
GGAATTGAATAGTTCAGAGCAACAAGATGCCATGCTACTGTGGTTAGAAGAACATGGTCGCCGTTTAGAACACGGCTTTATCAAACTTCATGATGAAGGA
AATGGCAAAAGCATTAATCTTTTCCCAGAAGAACCCCATATTTGTTCAACGGCTGTTACTAATGGTGTGAAGGTTCGCGCTTCTGCACTGGTTATCCCTG
AGTTGATGGATCTTCAAGATGACCTTGGAGAGTACTTATTTGCTTATTCAATCCGCTTGTCCCTTGAACCTCAAGGATGCATTATTAATGGAATGTCCTT
CAGCTCTTGCCAGCTCCATTGGAGGCACTGGATCATCCGTGCTAATGATATTGTTATATCTGATGTCAGTGGAAAAGCTGTTATAGGACAGTTTCCACTT
TTGCGTCCGGGTGCTCAAGAATTTGTTTATCAGAGTTGCACGCCTCTACCAACACCATCAGGTTCTATTGAAGGTTCTTTTACATTTATACCCGGCAGAT
TGGCAGACCCAAAAGGAGACCCTTTTCTAGCTACAGTGGCTCGTTTCCCGCTCCAGCTGCCAGACTATATATTCTGATTTTGATTCTGGATGGGATTGAA
GTATCTCTAATGGCAATTGCAGCTCTCAGATATTGTTTGGGTGTATTATGTCCCTTTCGGGAGAGGTTTTTAAGTTGGACCTGCACAATTTCCCTATCTA
TAAGAGAAGCAAAGTATGTACAATATTTGATCTCTATCATAAGAGAAGCAAAGTAGATACAATATTTGTTAGGGGTGTTCATGGTTTGATTAGGTTGGAT
TTTTTGTTAAAAAATCATCCAAATCAAACTTAAAAAACTTGTGATTTGGTTCGATTCAGTTTTCATTTAAAATAAAATTCAAATCAGACCAAGTTAATAT
ATATTGCACTTTAGTTCAA

Fbox, F-box protein family

>Glyma13g17830

ATTCATAGCTATTCGCAGTTCCCAAATTCATTTTAATCCTAATTCTGCACTTCTTGATCATTCGATTTTTCTCCTCAAGGTTCGTCTCTGTTTCATCTCT
TTTCATCATCGCAGCCTAATTAGCCTTAGTTTATTGATTTAGCTTTGGTCTTTCACCATGGTTACATTTTTACGTTTCTATCTTTCGATTTGTGATTTTT
TACGTTAATATGTGTATACCGGTTTTGTCGTTGTTGAATCCGAAACTTTCCATTCGACTTTCTGATTCCGTATCTCGTTTGTCACATGCAAAATCTTTCC
CGATTTTTAATCTGGCTATCTGATTTGATTTGATTTTGAGGAATTAGGGTTCGTGGTTTGATTTGATTCTGTGATTCGCTTGTTATATGTAAAATATTCC
CGGTTCCTAATTTTAGGGTCTGCGATTTATTTTCTTTTATCACTTTTGAGGAATCAGGGATCGTGGTCTGACTTATTGTGCATTATTTAGTTGTATGCTG
CAAAATCTTTCCTTATTTTTTAATTAGGGTTTAGGGTTTGCGATTTGTCATTTTTGGGAATTATGGTTTGTGGTTTATTGATTATGCAATTCGGGTTTGC
TTTCTTTCCGGTTTTTAATCAGAGTTCCTGACTTTGATGTTTCTTTTTTGTGATTGAACAGATGCAGATATTCGTAAAAACTTTGACCGGCAAGACCATC
ACCCTTGAGGTGGAATCCTCCGACACCATTGACAACGTAAAAGCTAAGATTCAAGACAAGGAAGGGATCCCACCTGACCAGCAGAGACTTATCTTTGCGG
GTAAACAACTTGAGGATGGTCGCACCCTTGCCGATTACAACATCCAAAAGGAATCAACCCTCCATCTCGTGCTTCGCCTCAGGGGTGGCATGCAAATCTT
TGTCAAGACTTTGACCGGGAAGACCATCACCCTTGAGGTGGAATCCTCGGACACCATTGATAATGTTAAGGCCAAGATCCAGGACAAAGAGGGCATCCCC
CCAGATCAGCAGAGGCTGATTTTTGCCGGTAAACAACTTGAGGATGGAAGAACCCTTGCTGATTACAACATTCAGAAGGAATCCACCCTTCACTTGGTGC
TTCGTTTGAGAGGTGGTATGCAGATTTTCGTTAAAACTTTGACCGGAAAGACCATAACCTTGGAGGTGGAGAGTTCAGATACCATAGACAATGTGAAAGC
TAAGATTCAGGACAAGGAAGGTATCCCACCAGACCAACAGAGGCTTATCTTTGCCGGGAAACAGCTTGAAGATGGAAGAACCCTTGCTGACTATAACATC
CAGAAGGAATCTACCCTGCACTTGGTGTTGCGGCTTCGTGGTGGCATGCAAATCTTTGTTAAGACTTTGACTGGAAAAACCATAACTTTGGAGGTTGAAA
GTTCTGATACCATTGATAATGTGAAGGCAAAGATTCAGGACAAGGAGGGCATTCCACCTGATCAGCAGAGGCTTATTTTTGCTGGGAAGCAACTGGAAGA
TGGTCGTACCTTGGCAGATTACAATATTCAGAAGGAATCCACCTTGCATCTCGTCCTTCGCCTTCGCGGTGGTTTTTAAGCGTGTTTTTTTTCAAGTGTT
CTTGTTTTAAGCGTGTCTTTTTTCAAGTGTTCTTGTATGTCATGTCTGTTATGTTCAATTTCTCCCTTGAACTTGTTTGCTGTTCTGGTTTAGGTGCTGC
TTTTTATGTTTTAATATGATGTATTGAGCTCGTTTCGCCAAAAGAATTAAATATCAATATCGTATCATAGTTAAATTCATATTTCGT

SUBI2, Ubiquitin

TUBa, Tubulin

```
>Glyma05g29000.1
AGGAGTATGTTAAGACTTGAGAGTGAGAGAGAGAGTGAGAGAGTGAGTGAGATAGAGAGAGACGCTTCGCAAATCTCAATCGAATCTCAGAAAAATGAGA
GAAATCATCAGCATTCACATAGGTCAGGCCGGGATCCAGGTCGGAAACTCCTGCTGGGAACTCTACTGCCTCGAACATGGCATCCAGCCCGACGGCATGA
TGCCTTCTGACTCCACCTTCGGTGTAGCCCACGACGCCTTCAACACCTTCTTCAGCGAAACCGGATCTGGCAAGCACGTCCCCCGTGCTGTCTTCGTCGA
CCTCGAACCCACCGTCATCGACGAGGTCCGCTGCGGCACCTACCGTCAACTCTTCCACCCCGAACAACTCATCTCCGGCAAGGAAGACGCCGCCAACAAC
TTCGCCCGCGGCCACTACACCGTTGGCAAAGAGATCGTAGATCTGTGCTTGGATCGCGTCCGCAAGCTCGCCGACAACTGCACCGGCCTACAAGGCTTCC
TCGTCTTCAACGCCGTCGGCGGTGGCACCGGTTCCGGTCTCGGATCTCTCTTACTCGAGCGTCTCTCCGTCGATTACGGCAAAAAATCCAAATTAGGGTT
CACCATTTACCCTTCCCCACAGGTTTCAACCGCAGTCGTTGAACCCTACAACAGCGTCCTCTCCACCCACTCCCTCCTGGAACACACCGACGTGGCGGTC
CTCTTGGACAACGAAGCCATCTACGACATCTGCAGGAGATCCCTCGACATCGAGAGACCAACCTACACCAACCTCAACAGGCTCATTTCCCAGATCATTT
CGTCCCTCACCACTTCCTTGAGGTTCGATGGTGCCATCAATGTGGATATCACTGAGTTCCAGACCAACCTTGTGCCCTACCCTAGGATCCACTTCATGCT
TTCGTCCTATGCTCCGGTTATCTCTGCCGCCAAGGCCTACCACGAGCAGTTGTCGGTGCCGGAGATCACCAATGCCGTGTTCGAGCCCGCCAGCATGATG
GCCAAGTGTGATCCAAGGCACGGCAAGTACATGGCTTGCTGCTTGATGTACCGTGGTGATGTTGTCCCTAAGGATGTCAATGCTGCTGTTGCCACCATCA
AGACTAAGAGGACTGTTCAGTTTGTTGATTGGTGTCCAACTGGATTCAAGTGTGGTATCAACTACCAGCCACCTTCTGTTGTTCCTGGTGGTGACCTTGC
TAAGGTTCAGCGTGCTGTATGCATGATCAGCAACAACACCGCAGTTGCTGAGGTGTTCTCTCGCATTGACCACAAGTTTGATCTGATGTATGCCAAGAGG
GCTTTTGTTCACTGGTATGTTGGTGAGGGTATGGAAGAAGGGGAGTTTTCTGAGGCTCGTGAGGACCTTGCTGCTCTTGAGAAGGACTATGAGGAGGTTG
GAGCTGAAGGAGCGGAAGATGATGAGGAAGGAGAGGACTATTGATAATCGAAGGCTTTTTTTGTTATCAATGTAATTGTTTCCGAGTTAAAAGGGTATA
TGCTTCGTTACATTACCCTGAACCATTATTGTTCGTTCTTTTTTGTTTTTGCTCCTGCTGTGTGATATAGTTCTCCATTATTCAAACTGTGAAGTGATCA
ACATTATGCTTCTATTTGGTGGTTTAGGACCTGTTGTTTGCGATCCTCAGTCATGTATTCAGAATTGTCCATGTCAAAAGTTGATTTCTTATGTTTATCT
CGCAAATTTTACCGGATTTCCCGTTTTGATTATCTCTTTATGAACCGTGAAAAACGTACCCCTCGTATGTTTTTTGCAATTCGCTTCATTCTGG
```

TUBb, Tubulin

```
>Glyma20g27280.1
GCTCACGCACCGTGAACCAACCCTGGTTACAGATAACCACAGCACAGCTACCCCATACATCTCCCTATAAAACCAATCCCAAATCCTACGAACGCTCCTC
ACTTCCAGATTTCAAAGAAGGCGTCTTCATAAACGCCTTTCTTCTCCGTTTCCGTTTCCTCGTTCGAATTCGCTTTTTGATCTGCGAAAATGAGAGAGTG
CATCTCGATCCACATTGGTCAGGCTGGTATCCAGGTCGGAAATGCTTGCTGGGAGCTCTACTGCCTCGAGCACGGCATCGGGCCCGATGGGCAAATGCCA
AGTGACAAGACAGTTGGCGGTGGTGATGATGCTTTCAATACTTTCTTCAGCGAGACTGGTGCTGGAAAGCACGTGCCACGTGCAGTGTTTGTAGATCTTG
AGCCCACTGTTATTGATGAGGTGAGAACCGGAACTTACCGCCAGCTTTTCCACCCAGAGCAGCTCATCAGCGGCAAAGAAGATGCCGCCAACAACTTTGC
CCGTGGCCATTATACCATTGGGAAAGAGATTGTTGATCTGTGCTTGGACCGCATCAGAAAGCTTGCTGACAATTGCACTGGACTCCAAGGGTTTTTGGTC
TTCAATGCCGTTGGTGGAGGAACTGGTTCTGGACTTGGTTCCCTTCTCTTGGAGCGTCTCTCCGTTGATTATGGCAAGAAATCAAAGCTTGGTTTCACAG
TCTACCCCTCTCCTCAGGTGTCCACCTCTGTTGTTGAGCCATACAACAGTGTCCTCTCCACCCATTCTCTCTTGGAGCACACCGATGTTGCTGTCCTCTT
GGACAATGAAGCCATTTATGACATCTGCAGACGCTCTCTCGACATTGAGCGTCCCACCTACACCAACCTCAACCGCCTTGTTTCTCAGGTGATTTCATCT
TTGACTGCTTCCTTGAGGTTTGATGGTGCCCTGAATGTGGATGTGACTGAATTCCAGACCAACTTGGTCCCATACCCTAGAATCCATTTCATGCTTTCCT
CCTATGCACCAGTTATCTCTGCTGAGAAGGCCTACCATGAACAGCTCTCAGTGGCAGAAATTACCAACAGTGCTTTTGAGCCATCGTCCATGATGGCCAA
GTGTGATCCTCGTCACGGAAAGTACATGGCGTGTTGCCTGATGTACCGTGGTGATGTTGTGCCCAAAGATGTAAACGCTGCTGTTGCCACCATCAAGACC
AAGAGGACCATTCAGTTTGTGGATTGGTGCCCTACTGGTTTCAAGTGTGGTATTAACTACCAGCCACCTACCGTTGTTCCTGGAGGCGACCTTGCCAAGG
TGCAGAGGGCAGTTTGCATGATTTCAAACTCCACCAGTGTGGCTGAAGTGTTTGGCCGCATTGATCACAAGTTTGATCTCATGTATGCCAAGCGTGCTTT
TGTCCACTGGTACGTGGGTGAGGGTATGGAAGAAGGTGAATTTTCTGAAGCCCGTGAGGACCTTGCAGCCCTTGAGAAGGATTATGAAGAAGTGGGTGCT
GAGTCTGGTGAGGGCCAGGAAGAGGATGAAGACTACTAGAATCTACTTCAGATTCTTGTTCTTGTGGTCTGTTTTCTGTTGTGTTTGATGTTCAGATGTG
TACCAGTACCGATGTGGTTTTAATTTGGATGCATTGCTTTTATGCTTCGCTGCTCCTGTTACGTACCTAAACTGAATGGCAATATTGAACTATGTTGTTT
TAGTTGTGTAATTGTGTTAATTGACTCATTACGGGCACGGGAGTGCGGCAATTGGTTAAGCAGCCTGATTTTGCTAAGGCAGTGGGTTATAGCAGCAGGG
TGGATTCATTAGTGGTACTTCTATGGAATTTAACTGGGCCTAAAATTTGACAAAAAATAAAACTCTTAAATGGTAGAGGGTTATAAACCTACAATAGAAA
AATCTGTTGATTTTGGAGTTTTAGTGATATTTTGTTGAGGAAAATTTTCTAAAACTCTTGTTCTAAATAGACCAGCGTAGCTATTTTAATAAAATTATAA
TACACTTTAGGGTTGCACAATTATAATACATAGTTATTGCAGAT
```
